# In silico docking of urokinase plasminogen activator and integrins

**DOI:** 10.1186/1471-2105-9-S2-S8

**Published:** 2008-03-26

**Authors:** Bernard Degryse, Juan Fernandez-Recio, Valentina Citro, Francescol Blasi, Maria Vittoria Cubellis

**Affiliations:** 1 San Raffaele Scientific Institute, Milan, Italy; 2 Life Sciences Department, Barcelona Supercomputing Center, Spain; 3 Dipartimento di Biologia strutturale e funzionale, Universita' di Napoli“Federico II”, Italy

## Abstract

**Background:**

Urokinase, its receptor and the integrins are functionally associated and involved in regulation of cell signaling, migration, adhesion and proliferation. No structural information is available on this potential multimolecular complex. However, the tri-dimensional structure of urokinase, urokinase receptor and integrins is known.

**Results:**

We have modeled the interaction of urokinase on two integrins, αIIbβ3 in the open configuration and αvβ3 in the closed configuration. We have found that multiple lowest energy solutions point to an interaction of the kringle domain of uPA at the boundary between α and β chains on the surface of the integrins. This region is not far away from peptides that have been previously shown to have a biological role in urokinase receptor/integrins dependent signaling.

**Conclusions:**

We demonstrated that *in silico* docking experiments can be successfully carried out to identify the binding mode of the kringle domain of urokinase on the scaffold of integrins in the open and closed conformation. Importantly we found that the binding mode was the same on different integrins and in both configurations. To get a molecular view of the system is a prerequisite to unravel the complex protein-protein interactions underlying urokinase/urokinase receptor/integrin mediated cell motility, adhesion and proliferation and to design rational *in vitro* experiments.

## Background

The serine protease urokinase-type plasminogen activator (uPA) and its high affinity cell surface receptor (uPAR) play an important role in a number of physiological as well as pathological extracellular degradation processes where cell migration is required, such as fibrinolysis, inflammatory responses and tumor invasion [1]. uPA is made up of three domains, the aminoterminal growth-factor-like domain, the kringle domain and the protease domain. The first two domains form the ATF (amino terminal fragment) a domain that binds uPAR [2] and whose structure has been solved by X-ray crystallography [3]. In the ATF it is the growth factor domain that binds uPAR. No real function has been established so far for the kringle domain.

uPAR is a heavily glycosylated GPI-ancored protein formed by three cysteine-rich LY6-like extracellular domains (D1,D2, and D3) connected by short linker regions [4]. The three domains of uPAR are organized in a bowl-like shape with a “fissure” between domains D1 and D3 and a deep central cavity for the interaction with the growth factor domain of uPA. The whole external surface of uPAR is available for other interactions [5].

Genetic and biochemical evidence shows that uPA and uPAR are involved not only in the regulation of fibrinolysis and cell surface-focused pericellular proteolysis [2], but also in the regulation of intracellular signaling affecting cell adhesion, migration, and proliferation [1,6-9]. Some, but not all, of these functions require the proteolytic activity of uPA.

Identified interactors of uPA/uPAR are trans-membrane signaling molecules: integrins, the G protein-coupled receptor FPRL1, the EGF-receptor (EGFR), the mannose-6-phosphate receptor, the family of low density lipoproteins receptor-related proteins (LRP), p130 and others [1]. The involvement of integrins was originally proposed on the basis of co-immunoprecipitation experiments in hematopoietic cells [10]. Both uPA and uPAR have since been reported to interact with cell adhesion receptors of the integrin superfamily, including subfamilies α1, α3, α5, as well as α2 expressed in cells of hematopoietic lineage and containing an I (insertion) domain [11]. More recently, also the β1 subunit has been proposed to participate in the interaction with uPAR [12].

The extracellular segments of the α- and β–subunits of integrins are up to 1104 and 778 residues long, respectively, with the N-terminal portions of each subunit combining to form a globular ligand-binding “head”. The structure of two such integrins is known, aIIbb3 in the open configuration and αvβ3 in the closed configurations [13,14]. Integrins bind an Arg–Gly–Asp (RGD) peptide sequence, the cell recognition site present in numerous adhesive proteins. The binding site of RGD on integrin αvβ3 has been identified by x-ray crystallography [15].

No definite information is available on the interaction of uPA or uPAR with integrins. However, three peptides were found that bound uPAR and prevented integrins function and uPAR-integrin co-immunoprecipitation. The first peptide belongs to the α subunit and is located in the w4 repeat of the β-propeller. Peptide α325 derived from α3β1 integrin and αM25 derived from αMβ2, were shown biochemically to directly bind uPAR, although at high concentration, and to affect integrin and uPAR functions [16,17]. Also in the β1 chain two stretches of amino acids (corresponding respectively to β1P1 and β1P2 peptides) completely inhibited uPAR-dependent cell adhesion to fibronectin, thus suggesting that they might interfere with the binding of uPAR to integrin α5b1 [12].

Despite this wealth of evidence indicating a direct interaction between uPAR and at least some integrins *in vivo*, evidence of a direct interaction in a purified system is lacking. Indeed, a soluble form of uPAR can be co-immunoprecipitated with purified α3β1 and α5 β1 integrins, but only in the presence of uPA [12,18]. uPA-dependent co-immunoprecipitation was also observed in some cell lines [19]. In conclusion, even though it is absolutely clear that uPAR and integrins regulate each other, a direct interaction between uPAR and integrins is not really demonstrated and might also be (at least in certain cases) mediated by uPA.

The ligand of uPAR, uPA, regulates cell migration, adhesion and the function of α_M_β2 integrin in cells expressing uPAR [20]. More recent evidence shows that the amino acid sequence linking the ATF to the protease domain of uPA can interact with the αv β3 integrin [21].

The interaction of uPA, uPAR and integrins is important since in uPAR Ko cells at least some integrins have been shown to be inactive [17,22]. Thus, the identification of the mechanisms of contact between these three molecules is important. Since the 3D structure of uPA, uPAR, ATF-uPAR complex, of the extracellular region of αvβ3 and αIIbβ3 [3,5,13,14,23,24] has been solved, it might be possible to exploit the available information to model these interactions. We have investigated the binding of the urokinase kringle to integrins *in silico*. We report that residues 113-123 of the kringle domain can be docked onto the integrin αIIbβ3 and αvβ3 in a position that is close to regions in both the α and β subunits, previously suggested to potentially interact with uPAR [12,16].

## Results and discussion

We docked the kringle domain of uPA on integrins *in silico*.

**Figure 1:**
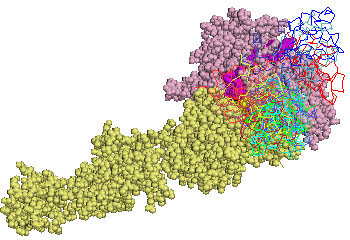
**Representation of the fifteen lowest energy poses of urokinase kringle domain on αIIbβ3 in the open conformation**. The α and β chains of αIIbβ3 are shown as pale pink and yellow spheres, respectively. The stretch of aminoacids homologous to the α325 peptide are highlighted in magenta, the stretch homologous to β1P1 in orange, and the one homologous to β1P2 in yellow. Ribbons represent every ligand positions after the rigid-body simulations. A spectrum of different shadows of red, green, blue, cyan were used going from lowest to highest energy solutions.

In our first experiment we docked the kringle domain onto a fragment of αIIbβ3 (residues 1-452 of the human αIIb chain, and residues 1-440 of the human β3 chain). This fragment represents the only possible scaffold of integrins in open conformation available in the PDB. We analysed the lowest energy solutions and in Fig.1 we arbitrarily chose to show the first 15. Eleven out of 15 solutions clustered together contacting the integrin at the boundary between the two chains. We have highlighted on the surface of the integrin scaffold three peptides which were reported to play a role in integrin-uPAR and/or integrin uPAR/ATF interaction in other integrins (no such information is available for the uPA interaction). The first peptide, which belongs to the α subunit and is located in the w4 repeat of the β-propeller [16,17], is coloured in magenta. Its homologues, peptide α325 derived from α3β1 integrin and αM25 derived from αMβ2, were shown biochemically to directly bind uPAR, although at high concentration, and to affect integrin and uPAR functions [16,17]. Two stretches of amino acids in the β chain are coloured in orange and in yellow and are homologous respectively to β1P1 and β1P2 peptides derived from the β chain of integrin α5β1.

A relevant biological result indicates that uPA is required to enhance co-immunoprecipitation of purified integrins and uPAR (see for example the paper by Degryse *et al*. [18]). These data thus indicate that the kringle domain could bridge uPAR and integrins. This is compatible with the location of the cluster of low energy solutions shown in Fig.1, which localises the kringle onto the peptides homologous to β1P1 and β1P2 not distant from the peptide homologous to α325 which might be spanned by uPAR.

**Figure 2:**
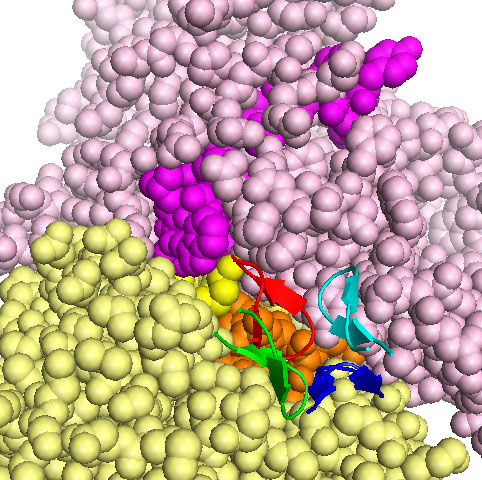
**Kringle tip contacts with αIIbβ3 in open conformation**. The α and β chains of αIIbβ3 are shown as pale pink and yellow spheres, respectively. The amino acids stretch homologous to the α325 peptide is in magenta, homology to β1P1 is in orange, homology to β1P2 in yellow. Res 113-123 are shown as cartoons, those corresponding to the second ranking solution are in red, those corresponding to the third ranking solution are in green, those corresponding to the fourth ranking solution are in blue and those corresponding to the fifth ranking solution are in cyan.

We also tested whether the same residues of the kringle domain in the complexes of the cluster were employed to contact integrin. Indeed, in 6 out 11 cases, it is the tip of the hairpin of the domain, residues 113-123 in active uPA, which contacts α2IIβ3. These solutions ranked 2nd, 3rd, 4th, 5th, 6th and 7th when the scoring function included electrostatic energy, desolvation energy and the van der Waals term. In Fig.2 a blow-up of the contact between the tips of four of the lowest energy solutions (2nd, 3rd, 4th, 5th) is shown, which highlights the closeness of the kringle residues 113-123 to both the α and β subunits peptides outlined above.

**Figure 3:**
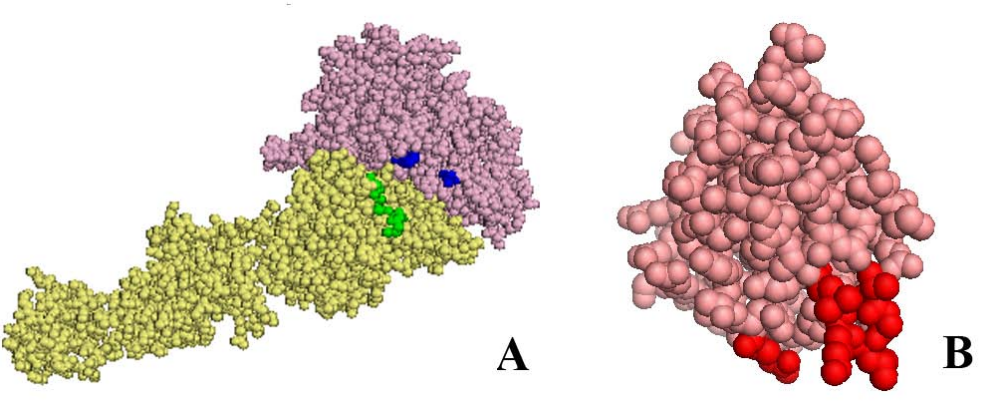
**Residues of αIIbβ3 in the open conformation and of urokinase kringle domain which are differentially exposed upon binding**. In panel A the α and β chains of αIIbβ3 are shown as pale pink and yellow spheres, respectively. Residues whose accessibility to the solvent changes by more than 10% upon kringle binding to αIIβ3 in the open form are highlighted in blue or green in the α chain or β chain respectively. In panel B the kringle domain of urokinase is shown as salmon spheres. Residues whose accessibility to the solvent changes by more than 10% upon binding onto αIIbβ3 in open form are highlighted in red. In any case, only the residues differently exposed in all poses ranking second, third, fourth and fifth are highlighted.

We identified the residues in the integrin α and β chain and on the kringle domain, which change their accessibility to solvent by more that 10% upon binding as seen in complexes ranking 2nd, 3rd, 4th and 5th. These residues mostly coincide in the different solutions. In Fig.3A, we show the residues of the open configuration integrin and in Fig.3B that of the kringle domain which undergo significative shielding from solvent in all analysed complexes (2nd, 3rd, 4th and 5th). They are highlighted in blue in the α chain, in green in the β chain and in red in the kringle domain. Although complexes generated by rigid body docking should be considered near native complexes because they are identified without taking into account induced fit, our results indicate that the relative orientation of the integrin and kringle domain in the low energy solutions is similar.

When we tried to dock uPAR, or uPAR in complex with growth factor domain, we did not observe a significant clustering of low energy solutions.

In uPA the catalytic serine protease moiety is preceded by a non catalytic amino-terminal fragment ATF. ATF binds the uPA receptor uPAR through its growth factor domain [2]. Visual inspection of the structure of ATF alone or in complex with uPAR reveals that the growth factor domain is a finger which fills an internal cavity of uPAR, formed by the interaction of the three domains, becoming almost completely embedded. The growth factor domain is connected by a flexible linker to the kringle, which stands as a structurally and functionally independent domain [3,23]. Although it is difficult to take into account the flexibility of the linker, we tried to dock the entire ATF-uPAR complex, in the conformation seen in the crystallographic structure 2i9b [3]. In nine out of ten lowest energy solutions we observed direct binding of the kringle domain to the α2IIβ3 integrin (not shown).Although the solutions were quite spread, in the first and fifth ranking solutions the kringle contacted α2IIβ3 at the border between α and β subunits trough the tip of the hairpin of the domain, residues 113-123, i.e.as observed when docking the isolated kringle to α2IIβ3 (Fig.2).

**Figure 4:**
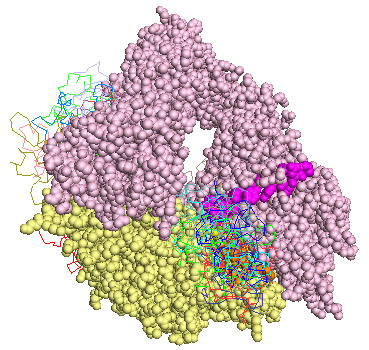
**Representation of the fifteen lowest energy poses of urokinase kringle domain on αvβ3 in the closed conformation**. The α and β chains of αvβ3 are shown as pale pink and yellow spheres respectively. The stretch of aminoacids homologous to α325 peptide are highlighted in magenta, homology to β1P1 is in orange, homology to β1P2 in yellow. Ribbons represent every ligand pose after the rigid-body simulations. A spectrum of different shadows of red, green, blue, cyan were used going from lowest to highest energy solutions.

**Figure 5:**
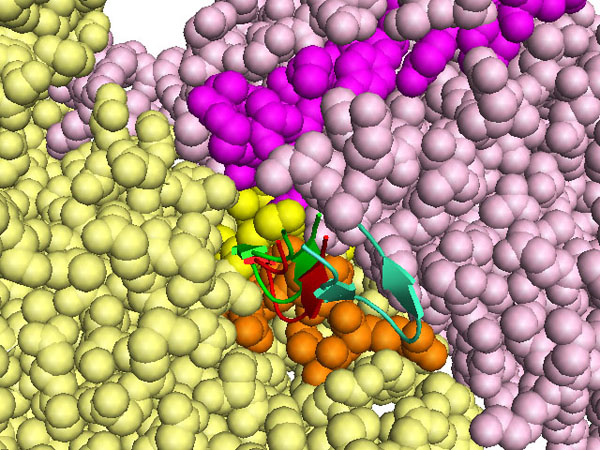
**Kringle tip contacts with αvβ3 in the closed conformation**. The α and β chains of αvβ3 are shown as pale pink and yellow spheres respectively. The stretch of amino acids homologous to the α325 peptide are in magenta, homology to β1P1 in orange, homology to β1P2 in yellow. Res 113-123 are shown as cartoons, those corresponding to the eighth ranking solution are in red, those corresponding to the ninth ranking solution are in green, those corresponding to the twelfth ranking solution are in blue.

We next carried out *in silico* binding of the kringle on the scaffold of an integrin in the closed configuration. The only structure available in PDB is that of αvβ3 (α chain residues 31-987, β chain residues, 27-718) [14]. The 15 lowest energy solutions clustered in two main groups (Fig.4). Solutions ranking 2nd, 6th, 8th, 9th, 12th, 13th and 14th when the scoring function included electrostatic energy, desolvation energy and the van der Waals term, localise in the same area already identified as a target of the kringle in the open configuration. In Fig.5 the stretches of amino acids homologous to α325, β1P1 and β1P2 peptides are coloured in magenta, orange and yellow respectively. In this case the inclusion of the biological data and the correspondence obtained docking the kringle domain on two conformations of the integrin is required to single out correct solutions.

**Figure 6:**
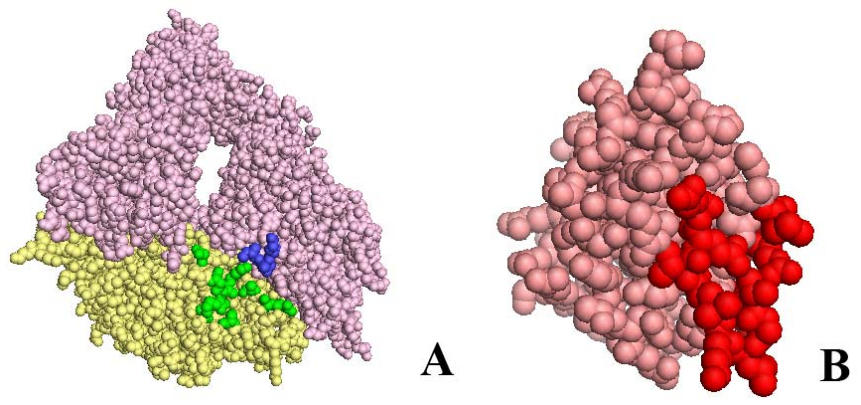
**Residues of αvβ3 in the closed conformation and of urokinase kringle domain which are differentially exposed upon binding**. In panel A the α and β chains of αvβ3 are shown as pale pink and yellow spheres respectively. Residues whose accessibility to the solvent changes by more than 10% upon kringle binding to αvβ3 in the closed conformation are highlighted in blue or green in the α or β chain, respectively. In panel B the kringle domain of urokinase is shown as salmon spheres. Residues whose accessibility to the solvent changes by more than 10% upon binding onto αvβ3 in the closed conformation are highlighted in red. In any case, only those residues differently exposed in the poses ranking eighth, ninth and twelfth are highlighted.

The same residues 113-123 of the kringle domain are likely to form the contact with the integrin in the open as well as in the closed form. This is seen in complexes ranking 8th, 9th and12th. Fig.6 shows a blow-up and the orientation of the kringle tip with respect to the stretches of amino acids homologous to α325, β1P1 and β1P2 peptides (coloured in magenta, orange and yellow). It is worth noting that complexes ranking 8th and 9th are almost superimposable. Complex ranking 12th when electrostatic energy, desolvation energy and the van der Waals term are included in the scoring function, ranks first when only electrostatic energy and desolvation energy are included. It has been reported that in some cases near native docking solution are better spotted excluding van der Waals energy terms from the scoring equation because this term is too sensitive to small structural perturbations [25].

The RGD binding sequence is located very close to the stretch of aminoacids corresponding to β1P1 peptide and to the interaction site with the kringle domain. Thus the kringle domain might affect the binding of RGD-containing substrates to integrins. Docking solutions can be considered only representative of near native complexes and do not allow to predict whether binding of uPA to the integrin would or would not be competitive with respect to RGD.

We have identified the residues on integrin αvβ3 α and β chains and on the kringle domain which change their accessibility to solvent by more that 10% upon binding, as seen in complexes ranking 8th, 9th and 12th. These residues mostly coincide in the different solutions. In Fig.6A we show the residues of the open configuration integrin and in Fig.6B that of the kringle domain which undergo significative shielding from solvent in all complexes analysed (8th, 9th and 12th). They are highlighted in blue in the α chain, in green in the β chain and in red in the kringle domain. Comparing Fig.3 and Fig.6, it should be noticed that the relative orientation of kringle and integrins appears to be the same in both open and close forms.

## Conclusions

Our studies strongly indicate that the kringle domain can mediate the binding of uPA to integrins. The kringle domain can bind intergrin in the open and closed conforation. This interaction does not appear to be in competition with the possible direct binding of uPAR to integrins, while it might possibly interfere with the RGD-dependent binding of the integrins to its substrates. Direct molecular studies will have to address this point. However, biological studies have already indicated that the kringle is essential in the pro-adhesive effect of uPA in cells that express α_M_β2 [20] and that truncated uPA without the uPAR-binding domain can bind integrin αvβ3 [21]. Our results are therefore strongly supported by these observations.

## Methods

The structure of human ATF/uPAR is deposited in pdb with the code 2i9b [3]. The structure was visually inspected to derive two domains: the first domain comprises uPAR (res 1-277), growth factor like domain (res11-42 of uPA) and kringle domain (res 43-132 of uPA).

In this paper uPA is numbered starting from first residue after signal peptide cleaveage.

For the integrin in open form we used the structure deposited with pdb code 1jv2. To solve this structure a fragment comprising residues 31-987 of the human αV chain, and residues 27-718 of the human β3 chain was crystallised [14].

For the integrin in closed form we used the structure deposited with pdb code 1txv. A fragment comprising residues 1-452 of the human αIIb chain, and residues 1-440 of the human β3 chain was crystallised [13].

Cofactors, ions and other heteroatoms were not considered.

To build models of integrin-kringle domain comples we used the suite of docking programs called pyDock [25]. Ten thousand rigid-body docking solutions are generated by the FFT-based programs FTDOCK [26] in each experiment. Then, the docking solutions are automatically evaluated with the module pyDockSER optimised for rigid-body docking landscapes, by the equation:

Ebind=Eele+Edes+WEvdw

where Eele is the binding electrostatics energy (Coulombic potential with distance-dependent dielectric constant e=4r, truncated to a maximum and minimum value of +1.0 and −1.0 kcal/mol, respectively) and charges from AMBER 94 force field [27]; Edes is the desolvation energy upon binding, based on atomic solvation parameters previously optimised for rigid-body docking. Evdw is the van der Waals binding energy based on the 6-12 Lennard-Jones potential, with atomic parameters from the AMBER 94 force field, truncated to a maximum of 1.0 kcal/mol to avoid much noise from the docking of rigid body surfaces; W is weight which was set to 0.1.

No spatial or biological restrictions were used during simulations, which allowed a complete sampling of the docking landscape around integrins

The residue solvent-accessible area were calculated using Naccess [28] with a 1.4 Å probe radius

Figures were drawn with Pymol [29].

## List of abbreviations used

uPA: urokinase type plasminogen activator

ATF: amino terminal fragment of the urokinase type plasminogen activator

uPAR: urokinase type plasminogen activator receptor

EGF: epidermal growth factor

FPRL1: formyl peptide receptor-like 1

LRP: low density lipoproteins receptor

GPI: Glycosylphosphatidylinisotol

## Competing interests

All the authors declare that they have no competing interests.

## Authors' contributions

MVC conceived the study and designed the experiments. JFR provided coding and discussion on the methodology. MVC carried out the experiments. MVC and FB wrote the manuscript. VC helped in the elaboration of data. BDG provided information about the biological system.
